# Inducing representational change in the hippocampus through real-time neurofeedback

**DOI:** 10.1098/rstb.2023.0091

**Published:** 2024-10-21

**Authors:** Kailong Peng, Jeffrey D. Wammes, Alex Nguyen, Coraline Rinn Iordan, Kenneth A. Norman, Nicholas B. Turk-Browne

**Affiliations:** ^1^ Department of Psychology, Yale University, New Haven, CT 06510, USA; ^2^ Interdepartmental Neuroscience Program, Yale University, New Haven, CT, USA; ^3^ Department of Psychology, Queen’s University, Kingston, ON, Canada; ^4^ Centre for Neuroscience Studies, Queen’s University, Kingston, ON, Canada; ^5^ Department of Psychology, Princeton University, Princeton, NJ, USA; ^6^ Princeton Neuroscience Institute, Princeton University, Princeton, NJ, USA; ^7^ Department of Brain and Cognitive Sciences, University of Rochester, Rochester, NY, USA; ^8^ Department of Neuroscience, University of Rochester, Rochester, NY, USA; ^9^ Wu Tsai Institute, Yale University, New Haven, CT, USA

**Keywords:** real-time fMRI, non-monotonic plasticity, closed-loop neurofeedback, high-performance computing, psychophysics, machine learning

## Abstract

When you perceive or remember something, other related things come to mind, affecting how these competing items are subsequently perceived and remembered. Such behavioural consequences are believed to result from changes in the overlap of neural representations of these items, especially in the hippocampus. According to multiple theories, hippocampal overlap should increase (integration) when there is high coactivation between cortical representations. However, prior studies used indirect proxies for coactivation by manipulating stimulus similarity or task demands. Here, we induce coactivation in visual cortex more directly using closed-loop neurofeedback from real-time functional magnetic resonance imaging (fMRI). While viewing one object, participants were rewarded for activating the representation of another object as strongly as possible. Across multiple real-time fMRI sessions, participants succeeded in using this neurofeedback to increase coactivation. Compared with a baseline of untrained objects, this protocol led to memory integration in behaviour and the brain: the trained objects became harder for participants to discriminate behaviourally in a categorical perception task and harder to discriminate neurally from patterns of fMRI activity in their hippocampus as a result of losing unique features. These findings demonstrate that neurofeedback can be used to alter and combine memories.

This article is part of the theme issue ‘Neurofeedback: new territories and neurocognitive mechanisms of endogenous neuromodulation’.

## 1. Introduction

Unlike a hard drive that stores files in dedicated blocks, the brain partially re-uses neurons that already store existing memories when forming a new memory. Because of the resulting overlap, when we later attempt to retrieve one memory, other related memories can come to mind. The competition between these coactivated memories can cause interference and behavioural errors in the moment. At the same time, such coactivation can also induce learning, with consequences for how the target and competitor memories are subsequently represented [1–3]. Namely, this learning can increase the amount of subsequent overlap between the neural populations representing each memory (integration), or it can reduce overlap (differentiation).

Both integration and differentiation have been reported at the level of distributed representations captured by functional magnetic resonance imaging (fMRI). Integration has been observed in statistical learning tasks in which arbitrarily chosen objects reliably co-occur in time or space [4–7]. For example, when participants view a continuous sequence of fractal images containing temporal pairs, in which one image is always followed by another, the paired images come to evoke more similar patterns of fMRI activity in the hippocampus [4]. Integration has also been observed in tasks involving deliberate associative learning: linking two arbitrary cues to a common associate increases the similarity of fMRI activity patterns for the two cues in the hippocampus and other regions [8–11]. Strikingly, closely related studies (in some cases, other conditions within the same study) have observed the exact opposite result of differentiation [4,11–17]. For example, pairing two cues with the same associate can sometimes lead to differentiation in the hippocampus [11,15].

Accounting for these discrepant findings poses a major theoretical challenge for the field of memory research. According to Hebbian plasticity [18–20], coactivation between memories should strengthen synaptic connections to and among the neural populations these memories share, resulting in integration that scales monotonically with the amount of coactivation. This diverges from the non-monotonic plasticity hypothesis (NMPH), which posits that representations change as a U-shaped function of coactivation between memories [3,21]: no coactivation leads to no representational change, moderate coactivation leads to differentiation, and high coactivation leads to integration. Prior attempts to study these theories have relied on stimulus similarity [14] or retrieval task instructions [11] to manipulate coactivation. Here, we develop a framework for manipulating memory coactivation more directly with closed-loop neurofeedback. As a first step, this study focuses on a point of agreement between Hebbian and NMPH theories of learning—that strong coactivation should lead to memory integration. If successful, this framework could be used to target moderate levels of coactivation to adjudicate between these theories.

Our approach is grounded in developments from the field of real-time fMRI over the past decade [22]. Real-time fMRI refers to conducting an fMRI study in which the blood oxygenation level-dependent (BOLD) data are analysed immediately after collection, such that the resultant estimates of brain activity can be provided as feedback to the participant or used to modify the task in a closed-loop manner [23,24]. This technique has found increasing success in clinical studies to modulate disordered brain activity, such as in depression [25,26], and for basic science discovery, to train or test theories of cognitive abilities such as sustained attention [27], perceptual learning [28], category learning [29] and episodic memory [30].

This study includes five fMRI sessions per participant: a pre-training session, three real-time neurofeedback training sessions and a post-training session. During the neurofeedback sessions, participants are trained to incept activation of a ‘competitor’ object (e.g. a chair) in visual cortex while another ‘presented’ object (e.g. bed) is being shown on the screen. To the extent that participants learn to strongly activate the competitor, we hypothesize that the representations of the presented and competitor objects will integrate. From pre- to post-training sessions, this integration should worsen behavioural discrimination of the objects in a categorical perception task and worsen neural discrimination when classifying patterns of hippocampal activity evoked by the objects. In addition to assessing change over time for the trained objects from pre- to post-training, we also include a baseline condition in these sessions consisting of two other objects (e.g. a table and a bench) that did not undergo neurofeedback training.

## 2. Methods

### (a) Participants

A total of 20 healthy young adults (mean age = 23.55; age range = 19−28; 14 female) with normal or corrected-to-normal visual acuity participated in this study. They completed five fMRI sessions lasting 1.5 hours each and were paid $20 per hour. Sessions were scheduled on separate days but as close together as possible, from a minimum of five days in a row to a total span of eight days. Two additional pilots and eight additional participants who did not complete all five sessions were excluded from the analysis.

### (b) Data acquisition

Data were acquired using a 3T Siemens Prisma scanner with a 64-channel head coil at the Brain Imaging Center at Yale University. For recognition and feedback functional runs, an echo-planar imaging (EPI) sequence was used to collect BOLD data (TR = 2 s; TE = 30 ms; voxel size = 3 mm isotropic; FA = 90°; IPAT GRAPPA acceleration factor = 2; distance factor = 25%), yielding 36 axial slices. Each recognition run contained 145 volumes, and each feedback run contained 176 volumes. Two field map volumes (TR = 5 s; TE = 80 ms; otherwise matching the EPI scans) were acquired in opposite phase encoding directions. For anatomical alignment and visualization, we collected a three-dimensional T1-weighted magnetization-prepared rapid acquisition gradient echo (MPRAGE) scan (TR = 2.5 s; TE = 2.9 ms; voxel size = 1 mm isotropic; FA = 8°; 176 sagittal slices; IPAT GRAPPA acceleration factor = 2), and a three-dimensional T2-weighted fast spin echo scan with variable flip angle (TR = 3.2 s; TE = 565 ms; voxel size = 1 mm isotropic; 176 sagittal slices; IPAT GRAPPA acceleration factor = 2).

### (c) Real-time system

After image reconstruction, the DICOM files were streamed in real-time to Milgram, a high-performance cluster mounted on the Siemens console. The RT-Cloud software package [31] was used for preprocessing and analysis of each image, with the results transmitted to the task computer at the scanner over the network. This output was used to update the task on the next time point.

### (d) Data preprocessing

For real-time analyses, motion correction was applied by aligning each new DICOM file with a template volume acquired from the middle of the first recognition run in the current session using 3dvolreg [32]. The BOLD activity of every voxel was normalized by *z*-scoring over time using the running mean and standard deviation from the prior TRs in the current run; this ensured that baseline differences in the mean or scaling of BOLD activity across voxels did not confound or bias classifier training. The data were masked to include only voxels in the region of interest (ROI) used for feedback prior to classifier training and testing. For offline analyses, the functional data were field map corrected with the topup tool in FSL [33], registered to the middle volume of the current run with MCFLIRT [34], and *z*-scored based on the full-time series from that run.

### (e) Study design

The study consisted of five sessions (table 1). Session 1 contained eight recognition runs in which the presented, competitor and control objects were shown repeatedly. These data were used to train classifier models that could distinguish between all pairs of objects. The trained models were then tested on the feedback runs in sessions 2, 3 and 4 to measure the amount of evidence for the competitor object during the viewing of the presented object. Participants were encouraged to increase the activation level of the competitor through adaptive, closed-loop neurofeedback, inducing coactivation between the presented and competitor objects. Session 5 mirrored the first session with eight recognition runs in order to assess representational change. We based the use of three neurofeedback sessions (and five total sessions) loosely on other real-time fMRI studies in our lab [26] and in other labs [35].

**Table 1.** Multi-session study protocol for each participant.

	session 1 (~1.5 h)	session 2 (~1.5 h)	session 3 (~1.5 h)	session 4 (~1.5 h)	session 5 (~1.5 h)
task order	categorical perception task	2 recognition runs	2 recognition runs	2 recognition runs	8 recognition runs
	8 recognition runs	10 feedback runs	10 feedback runs	10 feedback runs	categorical perception task
		2 recognition runs	2 recognition runs	2 recognition runs	

**Figure 1.**
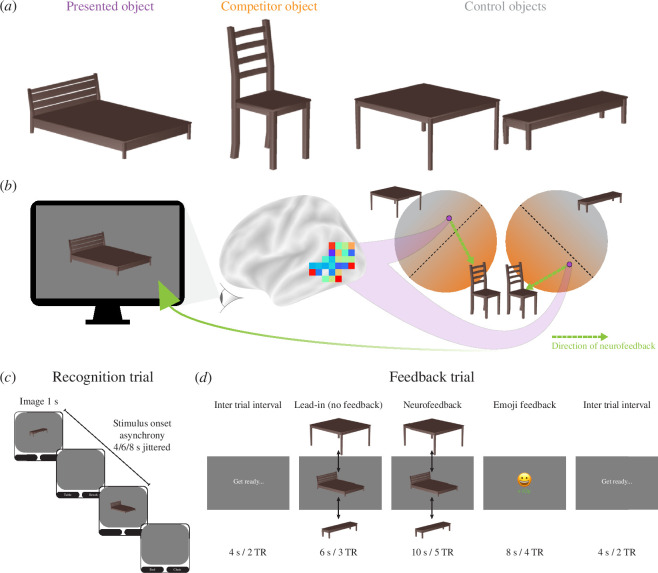
Study design. (*a*) Each participant received two objects for neurofeedback, and the other two objects served as a baseline. (*b*) The presented object (e.g. bed) was shown on neurofeedback trials and began oscillating in size and shape. The goal of the participant was to make this wobbling stop, which they could achieve by activating the representation of the competitor object (e.g. chair) in their mind. Evidence for the competitor object was quantified based on a classifier trained to decode the competitor object relative to two control objects (e.g. table, bench). The amount of classifier evidence for the competitor needed to reduce the magnitude of wobbling was staircased to maximize coactivation between these objects. (*c*) Recognition trials showed one of the four objects at a time with no neurofeedback. These trials were used to train the classifier models and to measure neural snapshots of how the object is presented in the hippocampus. (*d*) Feedback trials were conducted during real-time fMRI to induce coactivation. The main neurofeedback occurred during the object presentation (the amount of wobbling), though participants also received feedback at the end of the trial (monetary reward, valenced emoji). The real-time analysis of each test TR occurred during the acquisition of the next TR by transferring the reconstructed DICOM file instantaneously to a high-performance computing cluster. Because of the hemodynamic lag, we fixed the wobbling at the maximum level for a lead-in period of the first three TRs and began updating it on the fourth TR based on a running average of all TRs collected in the current trial up to that point.

This design employs a within-participant control condition rather than a separate sham or yoked control group. Specifically, one pair of objects served as the target of neurofeedback (trained) and the other pair of objects was a no-neurofeedback baseline (untrained). The assignment of pairs to conditions was counterbalanced across participants. For 10 of the participants (figure 1*a*), the bed (presented object) and chair (competitor object) were the trained pair, and the table and bench (control objects) were the untrained pair. For the remaining 10 participants, the assignments were reversed with table (presented object) and bench (competitor object) as the trained pair and bed and chair (control objects) as the untrained pair. By comparing behavioural and neural changes for trained versus untrained pairs, each participant serves as their own control when assessing the effects of neurofeedback. We based this within-participant control approach on prior neurofeedback studies of learning [28]. Such designs can be efficient because they avoid between-subject variability (e.g. individual differences, cohort effects) that can complicate group comparisons.

### (f) Recognition runs

During each trial of the recognition runs (figure 1*c*), participants were presented with one of four rendered furniture objects (bed, bench, chair, table) in one of several potential viewing angles [36,37]. After 1 s, the object was removed from the screen, and two furniture labels appeared below, from which participants had to choose which matched the object with an MRI-compatible button box. This response occurred during a jittered interval between trials of 4, 6 or 8 s. Each recognition run contained 48 trials, allowing 12 repetitions per object and run. Three repetitions of each object appeared in each quarter of a run (no back-to-back repetitions) to ensure an even distribution of objects over time.

The data from the recognition runs in session 1 were used to train six binary classifiers, corresponding to all combinations of the four objects. We used logistic regression classifiers with L2-norm regularization (penalty = 1). Each of the six classifiers contrasted one pair of objects (e.g. chair versus bench). The training data consisted of patterns of BOLD activity across voxels in a feedback ROI (described below) extracted 4 s after the onset of the object on each trial to account for the hemodynamic lag, labelled by the identity of the object. We validated that the amount of training data was sufficient by quantifying the sensitivity and specificity of the classifiers (electronic supplementary material, figure S1).

### (g) Feedback runs

During each trial of the feedback runs (figure 1*d*), the presented object was shown dynamically on the screen, appearing to wobble in size and shape [29]. BOLD activity patterns were extracted from the feedback ROI and supplied as input to the classifiers. To determine the activation level of the competitor object (figure 1*b*), we averaged the output of the two classifiers trained to discriminate the competitor object from each of the control objects (i.e. competitor versus control1; competitor versus control2). Similarly, the activation level of the presented object was determined by averaging the output of the two classifiers trained to discriminate the presented object from each of the control objects (i.e. presented versus control1; presented versus control2).

Note that we did not use a classifier trained to discriminate activation of the presented object from activation of the competitor object because we wanted separate estimates of the evidence for these objects. For example, if both objects were active (desirable coactivation), the output of this classifier would be at chance, but this result would also be obtained if neither object is active. Instead, we relied on the control objects as a neutral baseline. An alternative approach for obtaining separate estimates of presented and competitor activation could have been to use a multi-class classifier trained to discriminate activation patterns across all four objects. However, the presented object may still dominate the output of this classifier because it was the only object available perceptually. Indeed, a bias towards the presented object was another reason we did not use a binary presented versus competitor classifier for neurofeedback, as we suspected that the evidence would always favour the presented object. This is problematic because it may have resulted in a ceiling effect, rendering the classifier insensitive to the subtler fluctuations in competitor activation that were needed for training. We felt that pitting the competitor object against the control objects would place them on a more equal footing in that none were available perceptually.

Participants received multiple forms of feedback to help motivate them to increase the activation level of the competitor object and foster its coactivation with the presented object, including visual feedback via wobbling, monetary feedback via an increase in their bonus compensation, and valenced feedback via an emoji. If participants successfully raised the activation level of the competitor above an adaptive threshold, the magnitude of wobbling decreased: wobbling began each feedback trial (consisting of 5 TRs) at level 13 (maximal) and reduced to level 9 after 1 TR above the threshold, level 5 after 2 TRs above the threshold, and level 1 (minimal) after 3–5 TRs above the threshold. Participants also received a monetary reward and an emoji after the trial based on the final number of above-threshold TRs: 0 TRs means no money and an unhappy face; 1 TR means no money and a neutral face; 2–3 TRs means 5 cents bonus and a smiling face; and 4–5 TRs means 10 cents bonus and a laughing face. We combined multiple indicators of performance in order to provide participants with rich feedback that was both timely and accurate, and to make the task more fun and engaging.

The wobbling was produced by showing a series of morphed images on a spectrum from 1 to 100, where 1 is the object at one end of the morphing axis, and 100 is an object at the opposite end. The four magnitudes of wobbling reflected different ranges of morph values: level 13 changed the image linearly from morph 1 to 40 and back (or 100 to 60 and back); level 9 morph [1,28] (or [100, 72]), level 5 morph [1,16] (or [100,84]), and level 1 morph [1,4] (or [100,96]). The same number of steps were used for each level, resulting in the fastest motion for 13 and slowest motion for 1.

Participants were informed about these types of feedback and that the feedback depended on their performance in the task. However, they were not instructed that the feedback was based on competitor activation, nor were they instructed on what mental strategy to use. Instead, they were instructed to explore different strategies that seemed to improve feedback. After the study, participants completed a debriefing questionnaire.

The threshold used for determining feedback was adjusted dynamically using an adaptive staircase procedure (electronic supplementary material, table S1). The goal in using staircasing was to start participants at a difficulty level they could achieve at the beginning during their strategy exploration but then to increase difficulty across runs and sessions such that they would be incentivized to activate the competitor object more and more strongly as they gained control of the feedback. When participants exhibited poor performance, the threshold was decreased, giving them an opportunity to improve and catch up. Conversely, the threshold was increased to create room for further improvement when participants demonstrated higher levels of control.

### (h) Feedback region of interest

The BOLD activity patterns used to train and test the object classifiers were extracted from a data-driven ROI. To define this feedback ROI, we used the neuroSketch dataset [37], in which the same four objects were shown multiple times to other participants. Each of the 300 parcels in the Schaefer atlas [38] classified as gray matter by Freesurfer [39] were retained for further analysis. Individual classifiers were trained for each parcel, and their test performance was quantified using a leave-one-run-out methodology. The performance from each parcel was averaged across all 25 participants in the neuroSketch dataset, resulting in a ranking of parcel performance. To identify the set of parcels that yielded the best performance, we built a mega ROI, adding in the voxels from the top-*N* parcels and re-calculating test performance for each value of *N* using a leave-one-run-out approach. The mega ROI composed of the 78 highest-performing parcels yielded the best object classification performance in the neuroSketch dataset.

**Figure 2.**
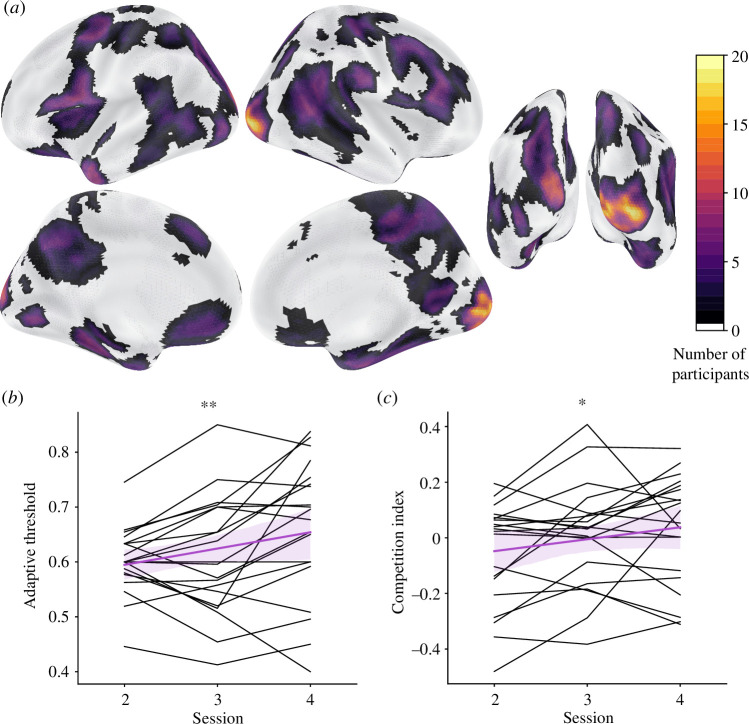
Neurofeedback validation. (*a*) The mega ROI used to provide neurofeedback was customized for each participant in a data-driven manner. This plot shows the number of participants whose feedback ROIs contained each voxel. The greatest consistency (virtually every participant) was observed in the early visual cortex, extending ventral and lateral. (*b*) Participants succeeded in self-generating classifier evidence for the competitor object based on neurofeedback, as indicated by an increase across the feedback sessions in the threshold from the staircasing procedure. A higher threshold over time indicates that participants were able to activate the competitor object more and more strongly. Black lines depict individual participants, the purple line depicts the linear fit combining across participants, and the light purple band indicates the 95% confidence interval of the linear fit. (*c*) As a corollary of this effect, we also found an increase across feedback sessions in the coactivation between the presented and competitor objects, quantified as the product of the classifier evidences for these objects relative to the control object baseline. ** p<0.01, * p<0.05.

This mega ROI with 78 parcels served as the starting point for each participant in the current study but was further refined per individual through a greedy approach. We first removed the voxels from one parcel (77 parcels remaining) and trained and tested a four-way classifier on the recognition runs from session 1 with leave-one-run-out cross-validation, and then iterated until all 78 parcels had been the one parcel left out; the iteration in which the remaining 77 parcels yielded the highest decoding performance was retained. Then we repeated the whole procedure, dropping one parcel (76 parcels remaining), iterating until all 77 remaining parcels had been left out, and then retaining the best set of 76 parcels. This process repeated until the voxels from only one parcel remained, yielding performance values for mega ROIs containing 1−78 parcels; the mega ROI with the best performance of all of these combinations was used as the feedback ROI for this participant. As a result, different participants had a different number of parcels in their mega ROI. However, there was good consistency in which parcels were selected, especially in the visual cortex (figure 2*a*).

Our design does not require a control ROI for sham or other feedback. We are not making a claim about which brain region(s) contain the most useful activity for neurofeedback. Instead, our conclusions pertain to the impact of neurofeedback training on representational change in the hippocampus and categorical perception in behaviour based on the within-participant comparison between trained and untrained pairs. Thus, the hippocampus and other ROIs were used to measure the neural outcomes of learning.

### (i) Representational change regions of interest

To examine how cortical coactivation related to representational change in the hippocampus, we segmented the hippocampus and its subfields anatomically for each participant using the automatic segmentation of hippocampal subfields (ASHS) software package [40] and a reference library of 51 manual segmentations [41,42]. These segmentations defined participant-specific ROIs for the bilateral hippocampus and subfields CA1, CA2/3, dentate gyrus and subiculum. We also explored broader effects in the cortex by using Freesurfer [43] to create masks for V1, V2, lateral occipital (LO) cortex, inferior temporal (IT) cortex, fusiform gyrus (FG) and parahippocampal cortex (PHC).

### (j) Representational change analysis

We assessed representational change in the primary hippocampal ROIs and the exploratory cortical ROIs by comparing the overlap of neural patterns for the presented and competitor objects in session 1 versus session 5 (using the same metric for control objects 1 and 2 as a baseline). The hippocampal ROIs were outside of the (cortical) mega ROIs used for neurofeedback. The cortical ROIs (V1, V2, LO, IT, FG, PHC) partially overlapped with the mega ROI in some cases. The use of some of the same cortical data from session 1 to assess representational change was not circular because the key dependent measure is the difference between trained and untrained objects, and the selection of the mega ROI was blind to which objects would be trained or untrained. Moreover, the cortical ROIs were defined *a priori* based on anatomy, and thus the selection of the mega ROI did not impact which cortical ROIs we analysed or bias the results of these analyses.

For session 1, we built eight regularized logistic regression classifiers for each participant and ROI, each using seven of the recognition runs for training, and the remaining recognition run for testing in a leave-one-run-out manner. The performance of each classifier was quantified using the area under the receiver operating characteristic (ROC) curve.

The ROC curve plots the true-positive rate (TPR, or sensitivity) against the false-positive rate (FPR, or 1-specificity) to illustrate the diagnostic ability of a binary classifier at various thresholds. TPR is defined as TPR=TP/(TP+FN), where TP denotes the true positives and FN denotes false negatives. FPR is defined as FPR=FP/(FP+TN), where FP denotes false positives and TN denotes true negatives. The area under the curve (AUC) for the ROC quantifies the classifier’s overall ability to distinguish between positive and negative outcomes, ranging from 0.5 (no discrimination) to 1.0 (perfect discrimination). The AUC for each classifier was computed using the ‘roc_auc_score’ function from Python’s sklearn package [44], which requires the classifier’s probability outputs and the actual labels.

For session 5, we tested the eight trained classifiers from session 1 on the eight recognition runs and averaged the AUCs to obtain an overall performance score for each participant. A decrease from session 1 to session 5 indicates reduced discriminability from greater neural overlap. We thus defined a neural integration index for each ROI as the average session 1 AUC minus session 5 AUC.

### (k) Geometric analysis of shared versus unique features

We decomposed this measure of overall representational change into the neural features shared between the presented and competitor objects and the neural features unique to each object (and repeated this analysis for control objects 1 and 2 as a baseline). Neural integration (i.e. a reduction in AUC) could reflect an increase in the expression of shared features and/or a decrease in the expression of unique features. We tested both possibilities by comparing how hippocampal patterns of activity evoked by the presented and competitor objects in the recognition runs (and control objects 1 and 2) changed from session 1 to session 5. For each participant and recognition run, we mean-centred the timecourse of activity in each voxel from the primary hippocampal ROI then calculated the average voxel pattern across all trials and recognition runs for each of the four objects, resulting in four voxel-length vectors.

To identify shared and unique dimensions for each participant, we normalized the vectors for the presented and competitor objects to unit length. The shared direction was identified as the sum of the presented and competitor objects (intuitively, a new vector halfway between the two unit vectors). The unique direction was identified as the difference of presented and competitor objects (intuitively, a new vector orthogonal to the sum, connecting the two unit vectors). We then projected the un-normalized vectors from sessions 1 and 5 onto these two orthogonal directions and calculated the difference in projections for session 5 minus session 1. We repeated this procedure for control objects 1 and 2, including defining their own shared and unique directions and then calculating the projections on these directions across sessions.

Note that we used the session 1 data to identify the shared and unique directions and then to calculate the pre-training projections. This was necessary because, by definition, the shared features need to be estimated from the initial representations themselves. If measured from other trials or participants, there would be no guarantee that these were the actual shared and unique features. Critically, any bias induced by this procedure applies equally to both the trained axis (presented and competitor objects) and the untrained axis (control objects 1 and 2), as these axes were treated identically in session 1. Thus, in the analyses below, the change in shared and unique projections for the trained axis is compared with the change for the untrained axis as an important baseline.

We quantified the change in shared features for each participant as the session 5 minus session 1 difference in projections on the shared direction for the trained axis minus the equivalent change for the untrained axis; a positive value indicates a relative increase in shared features. We likewise quantified the change in unique features as the session 5 minus session 1 difference in projections on the unique direction for the trained axis minus the equivalent change for the untrained axis; a negative value indicates a relative decrease in unique features. Using this vector geometry, we recomputed the overall neural integration effect in the hippocampus for each participant as the sum of the magnitude of increase in the shared direction and decrease in the unique direction.

### (l) Categorical perception task

To assess the impact of representational change on behaviour, we conducted a categorical perception task [29,36] before fMRI data collection in session 1 and after fMRI data collection in session 5. During this task, participants categorized images sampled from along a morph continuum between two object endpoints (e.g. bed to chair). Because the objects were rendered from three-dimensional models with a matching number of vertices, it was possible to smoothly morph between them. The morph percentage (of the second object) was sampled at 13 steps: 18 (i.e. 18% chair, 82% bed), 26, 34, 38, 42, 46, 50, 54, 58, 62, 66, 74 and 82. These morphs were shown 12 times each during both the pre-test and post-test, always from a trial-unique viewpoint. On each trial, participants were briefly presented (1 s) with the morph and asked to make a forced-choice judgement about which object they saw by clicking one of two buttons that appeared below the image. The assignment of labels to left versus right buttons was randomized across trials. A logistic regression model was used to analyse the relationship between the morphing parameter and categorization responses:


$$p(x)=\frac{1}{1+e^{-(x-\mu )/s}}$$

For the session 1 categorical perception task, the slope and μ parameters were estimated. In session 5, the μ value from session 1 was fixed (to reduce noise and enhance model capability), and we estimated the slope. The change in slope indicates the type and degree of representational change between the two objects being discriminated. In particular, a decrease in slope (reduced discriminability) when comparing the presented and competitor objects would be consistent with the integration of their representations, whereas an increase in slope (improved discriminability) would be consistent with differentiation. We thus defined a behavioural integration index as the session 1 slope minus session 5 slope (positive for integration, negative for differentiation). Importantly, these changes can be evaluated with respect to the analogous change observed for the untrained control objects.

### (m) Brain–behaviour relationship

To the extent that categorical perception is a behavioural readout of neural overlap in one or more ROIs, the behavioural and neural integration scores should be positively correlated across participants. We quantified this association in each ROI by calculating the Pearson correlation coefficient.

### (n) Statistics

We used non-parametric statistics where possible to avoid assumptions of parametric tests. To estimate the sampling distribution of an effect, we performed bootstrap resampling at the group level. Namely, from the original sample of 20 participants, for each of 1000 iterations, we sampled 20 participants with replacement and averaged their values. The mean and 95% bounds of the resulting sampling distribution were used to generate the bar plot. For hypothesis testing, we determined the *p*-value as the proportion of iterations on which the average had the opposite sign from the original effect.

## 3. Results

### (a) Validation of real-time neurofeedback

We sought to use real-time fMRI neurofeedback to generate coactivation between an object being perceived (presented object) and an object being internally activated from memory (competitor object). We tested whether participants succeeded in using neurofeedback to activate the competitor object in the feedback sessions (sessions 2−4) in two ways. First, we examined how the adaptive threshold from the feedback staircasing procedure changed over time; an increase in the threshold would indicate that participants were able to activate the competitor object more and more strongly. Indeed, using linear regression we found a reliable increase in the adaptive threshold across feedback sessions (figure 2*b*, p=0.004). Second, we more directly tested whether coactivation between the presented and competitor objects increased over time. We quantified coactivation by multiplying the classifier evidence from the feedback ROI for the presented object (relative to control objects) by the classifier evidence for the competitor object. Again, we found a significant rise in this neural ‘competition index’ over feedback sessions (figure 2*c*, p=0.025). Importantly, these analyses of the feedback ROI were performed on data (sessions 2−4) that were independent of the data (session 1) used to define the ROI.

### (b) Behavioural integration

**Figure 3.**
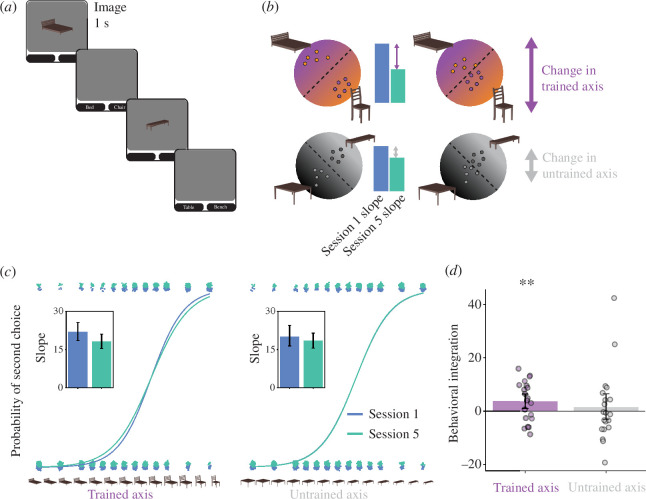
Categorical perception. (*a*) On each trial of the categorical perception task, participants were shown a morph on a continuum between two objects and asked to indicate via forced-choice responses which of the two objects they perceived. (*b*) Behavioural categorization responses can be modelled with logistic regression as a function of morph level. For each participant, separate models were fit to the first (session 1) and last (session 5) session and to discrimination of trained objects (presented versus competitor) and of control objects (control 1 versus 2). (*c*) The fitted slope parameters (see insets) were then used to calculate a behavioural integration index of session 1 minus session 5. (*d*) This index was reliably positive for the trained axis, reflecting a shallower slope after neurofeedback indicative of integration. However, there was no reliable change for the untrained axis, suggesting that neurofeedback was necessary for integration. Each dot represents an individual participant and the error bands reflect the 95% confidence interval from bootstrap resampling. ** p<0.01.

We next examined the impact of this induced coactivation on the representations of the presented and competitor objects from before (session 1) to after (session 5) neurofeedback training. We used a categorical perception task [36] as a behavioural assay of the overlap between representations. Participants were shown a morph of two objects and had to indicate which of the two objects they saw by selecting the label (figure 3*a*). To the extent that coactivation led to integration, greater reliance on the shared features of the two objects should make it harder for participants to discriminate the morph and lead to a shallower logistic slope; alternatively, if differentiation occurred, then greater reliance on the unique features of the two objects should exaggerate those features when perceiving the morph and lead to easier discrimination and a steeper slope.

A logistic regression was fitted to the categorization responses as a function of the morphing parameter (figure 3*c*), separately for sessions 1 and 5, and for the trained (presented versus competitor objects; e.g. bed, chair) and untrained (control 1 versus 2 objects; e.g. bench, table) axes. We calculated a behavioural integration index as the difference in the slope for session 1 minus session 5 (figure 3*b*); positive indicates that the slope got shallower. This index was reliably positive for the trained axis (p=0.009; figure 3*d*) but not for the untrained axis (p=0.314); the difference in indices between trained and untrained axes did not reach significance (p=0.242). This provides behavioural evidence of integration between the presented and competitor objects as a result of neurofeedback.

### (c) Neural integration

**Figure 4.**
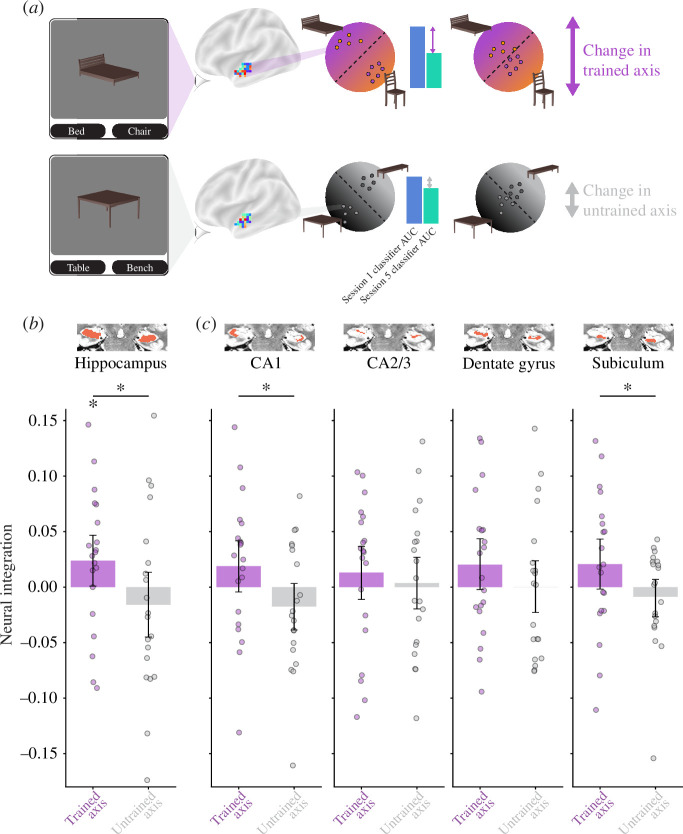
Representational change in the hippocampus. (*a*) We collected neural snapshots of how the four objects were represented in each ROI before (session 1) and after (session 5) neurofeedback training. Participants viewed the objects (intact, no wobbling or morphing) during a recognition task in these sessions. For each participant, we built regularized logistic regression classifiers to discriminate multivoxel patterns of BOLD activity in each ROI evoked by the presented versus competitor objects (trained axis) and by the control 1 versus 2 objects (untrained axis). Using leave-one-run-out cross-validation, we quantified the neural overlap along these two axes from the test classifier area under the curve (AUC). A neural integration index was defined for each ROI and axis as AUC in session 1 minus session 5 (positive reflects a decrease in AUC or reduced discriminability). (*b*) In the whole hippocampus, there was reliable neural integration along the trained axis relative to the untrained axis. The integration for the trained but not untrained axis was reliably above chance. (*c*) The difference in neural integration between trained and untrained axes was reflected most clearly in the CA1 and subiculum subfield. The insets depict the location of the ROI in red on an example participant’s T2 scan. Each dot in the bar plots represents an individual participant and the error bands reflect the 95% confidence interval from bootstrap resampling. * p<0.05.

We hypothesized the coactivation of object representations in the cortical inputs to the hippocampus would lead to representational changes in the hippocampus. According to the NMPH, strong coactivation should lead to integration, whereas moderate coactivation should lead to differentiation. To examine these representational changes, we applied binary classifiers to patterns of BOLD activity from hippocampal ROIs before (session 1) and after (session 5) neurofeedback training using cross-validation (figure 4*a*). If two objects integrate, their greater neural overlap should lead to misclassifications and reduced AUC; in contrast, differentiation and lower overlap should make classification easier and increase AUC. We calculated AUC from the ROC curve in the recognition runs from sessions 1 and 5, separately for the trained axis (presented vs. competitor) and untrained axis (control 1 versus 2). As with behavioural integration, we calculated a neural integration index for each ROI as the classification AUC in session 1 minus session 5 (positive indicates classification worsened).

In the overall hippocampus (figure 4*b*), there was a significant difference between indices for the trained and untrained axes (p=0.041); the trained axis (p=0.042), but not the untrained axis (p=0.820), was reliably positive. These results indicate that integration occurred as a result of neurofeedback in the hippocampus. We next investigated how these results were distributed across subfields within the hippocampus. This was an exploratory analysis for which we did not correct for multiple comparisons, as these tests were not independent of the overall hippocampal results. We were especially interested in the CA1 subfield, which has previously been linked to integration [11,45]. Indeed, the pattern from the broader hippocampus of a difference in neural integration between trained and untrained axes was reflected most clearly in the CA1 (p=0.023) and subiculum (p=0.021) subfields; none of the other subfields reached significance (ps>0.175). Neither the trained axis (ps>0.067) nor the untrained axis (ps>0.396) on their own significantly differed from chance in any subfield.

In the cortical ROIs, the PHC mirrored the hippocampus in showing integration as a result of neurofeedback, with a significant difference in neural integration index between trained and untrained axes (p=0.002), a reliably positive index for the trained axis (p=0.004), and no difference from chance for the untrained axis (p=0.949). None of the other cortical ROIs showed a difference in neural integration indices between trained and untrained axes (ps>0.148).

### (d) Geometric analysis

**Figure 5.**
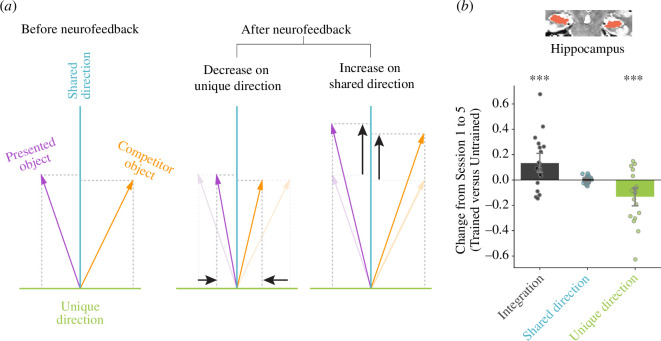
Geometric analysis of shared and unique features in the hippocampus. (*a*) Schematic illustration of the vector methodology for identifying shared and unique features in representational space. The presented (purple) and competitor (orange) vectors indicate the average patterns of activity in the hippocampus elicited by these objects, respectively, for one participant. Each voxel making up the pattern is a dimension in the representational space and the activity of that voxel is the coordinate value on that dimension. Before neurofeedback (recognition runs of session 1), the shared direction (blue) for each participant was calculated as the vector sum of the normalized presented and competitor vectors; the unique direction (green) was calculated from the same data as the (orthogonal) vector subtraction of the normalized presented and competitor vectors. After neurofeedback (recognition runs of session 5), a pure increase in the expression of shared features would grow the projection of the presented and competitor vectors on the shared direction without changing their projection on the unique direction; a pure decrease in the expression of unique features would conversely reduce the projection of the presented and competitor vectors on the unique direction without changing their projection on the shared direction. These analyses were repeated with the untrained axis (control objects 1 and 2) to provide a baseline. In principle, either or both of these changes, for the trained relative to the untrained axes, could be responsible for neural integration. (*b*) We first replicated the main neural integration effect in the hippocampus by summing the increase of shared projection and (absolute) decrease of unique projection from session 1 to 5, for trained versus untrained axes. This overall effect was driven by a reliable decrease of the unique projection, without a change in the shared projection. *** p<0.001.

The neural integration observed in the hippocampus could reflect an increase in the expression of features that were shared between the representations of the presented and competitor objects and/or a decrease in the expression of features that were unique to each representation (figure 5*a*). To isolate these changes, for each participant, we identified shared and unique directions in the voxel-dimensional representational space of their overall hippocampus in session 1 and examined the change in how neural patterns from session 5 projected onto these directions. We repeated this analysis for the untrained axis as a baseline.

Using this vector-based geometric approach, we replicated the main neural integration effect in the overall hippocampus as the sum of the magnitude of increase in shared features and decrease in unique features for the trained relative to untrained axes (p<0.001). Considering these two types of change separately (figure 5*b*), there was a highly reliable decrease in the expression of unique features for trained versus control axes (p<0.001), but no reliable increase for shared features (p=0.363). This analysis indicates that the hippocampal integration we observed reflected the pruning of unique features, perhaps because the unique features of the competitor object were harder to activate under competition from the presented object.

### (e) Relationship between behavioural and neural integration

Given that we observed both behavioural and neural integration after neurofeedback training, we next asked whether these effects were related. Namely, we tested whether the increased neural overlap of the trained objects observed in the hippocampus and PHC had behavioural relevance for categorical perception. There was a significant positive correlation between neural integration in PHC and behavioural integration (r=0.637, p=0.003), but not for the hippocampus (r=0.334, p=0.150). We repeated this analysis for the untrained objects as a control, and as expected, neither PHC nor hippocampus showed a reliable correlation (ps>0.105).

## 4. Discussion

The current study used closed-loop neurofeedback from real-time fMRI to provide a direct test of how the coactivation of object representations in the visual cortex drives representational change in the hippocampus. The key findings are that: (i) participants succeeded in using implicit neurofeedback to increase activation of the cortical representation of the competitor object while viewing the presented object; (ii) neurofeedback training reduced behavioural discrimination of this trained pair of objects but not a control pair of objects in a categorical perception task; (iii) neurofeedback training also reduced neural discriminability of the trained objects in the hippocampus, CA1, subiculum and PHC, relative to the control objects; (iv) neural integration in PHC was correlated with behavioural integration across participants; and (v) neural integration in the hippocampus was driven by a decrease in unique features of the trained objects, relative to the control objects. These results indicate that increasing coactivation of objects in the visual cortex can drive their neural representations in the medial temporal lobe to integrate by losing unique features and that this can have behavioural consequences for perception.

By encouraging participants to activate the competitor objects more and more strongly with a staircasing procedure and through additional feedback and monetary incentives, the coactivation levels of the presented and competitor objects may have been high enough to foster synaptic strengthening between their neural features. At a population level, such plasticity may increase the overlap of fMRI activity patterns evoked by the trained objects when presented individually in recognition runs from before to after neurofeedback training. This neural integration based on strong coactivation is predicted by both Hebbian learning [18–20] and the NMPH [3,21].

Distinguishing non-monotonic (NMPH) and monotonic (Hebbian) learning would have required exploring a broader range of coactivation levels under which these theories make different predictions. Namely, NMPH predicts that moderate coactivation would lead to synaptic weakening between the features of the presented and competitor objects, decreasing the similarity of fMRI activity patterns from before to after neurofeedback training (differentiation). In contrast, Hebbian learning predicts that moderate coactivation would still lead to synaptic strengthening and integration, just to a lesser degree. Future studies could employ different feedback regimes and/or stimuli to aim for more moderate coactivation. For example, rather than trying to maximize competitor activation through ever-rising staircasing and incentives, the amount of competitor activation could be titrated into a moderate band. Different groups of participants, or even different object pairs within-participant, could target different activation bands (e.g. low, medium, high) to better cover the hypothetical curve. Alternatively, our same feedback approach could be used but with pairs of objects with lower similarity in colour, shape and category; this reduced baseline similarity may make it more difficult to strongly activate the competitor object while perceiving the presented object, resulting in moderate competitor activation and possibly leading to differentiation [14]. It may also be possible to induce differentiation by changing the basis of the neurofeedback. By quantifying competitor activation relative to the control objects, we may have encouraged participants to activate features shared with the presented object (which was visible on the screen) and thus favoured integration; basing neurofeedback instead on the amount of competitor activation relative to the presented object may have encouraged them to activate unique features of the competitor object and thus favoured differentiation.

Our findings from real-time fMRI complement previous offline fMRI studies of representational change in the hippocampus [11,13–15,46]. These studies used a stimulus or task manipulation to achieve coactivation between competing memories, such as by increasing stimulus similarity [14] or by encouraging retrieval of related experiences [11]. We directly manipulated coactivation by using neurofeedback to incept a competing memory. Following the logic of prior real-time fMRI studies [28], this demonstrates that neural coactivation is sufficient for hippocampal integration. This is a stronger and more causal test of the hypothesis that neural competition drives representational change.

The role of the hippocampus, and CA1 in particular, in memory integration accords with existing findings [11] and computational models [45]. CA1, which receives cortical input from the entorhinal cortex along the monosynaptic or temporoammonic pathway, is a key site for integration because it has lower inhibition between neurons, which allows more neurons to become active and increases baseline overlap across memories. As a result, CA1 is more likely to enter a regime in which a target and competitor memories are active enough to strengthen their synaptic connections, resulting in integration at a population level. PHC, a key relay between the visual cortex and the hippocampus, has also previously been implicated in memory integration [47] and here was the only region to show a reliable brain–behaviour correlation. Based on studies showing that patients with hippocampal lesions but intact parahippocampal cortex fail to show behavioural integration, it is unclear whether PHC is sufficient [48]. We also observed a neural integration effect in the subiculum, a structure traditionally considered a relay from the hippocampus to other brain regions [49]. For example, the subiculum links to the brain‘s reward systems, which may have been engaged by the neurofeedback received [50]. An exciting direction for future research could be to examine how reward processing, for example, in the ventral tegmental area (VTA) [51] and ventral striatum [52–54], can modulate coactivation in visual cortex and representational change in the hippocampus.

In sum, this study employs real-time neurofeedback to induce memory competition and drive representational changes in the hippocampus. This approach may be more efficient than using predefined stimuli or tasks to manipulate competition, as it personalizes neurofeedback to the most proximal variable of interest—cortical activation of the competing memory. With further development and more portable and affordable neural recording technologies, this approach could find a variety of applications. As a technique for modifying specific memories, it could be used to develop innovative treatments for a range of neurological and psychiatric disorders. Daily functioning and quality of life could be enhanced in individuals suffering from age-related memory decline or memory impairments due to stroke by creating interventions to strengthen interconnected memories, such as associating the members of a category or re-linking the names of loved ones with their faces. The emotional impact of traumatic memories in PTSD could be reduced by integrating these memories with positive or rewarding experiences. Beyond healthcare, the ability to modify specific memories could be used for classroom education or occupational training to accelerate the learning of concepts and skills.

## Data Availability

The fMRI data are available here: https://doi.org/10.5061/dryad.kd51c5bg2. The code for analyzing these data can be found here: https://github.com/KailongPeng/real_time_neurofeedback. Supplementary material is available online [55].
